# The Modulatory Activity of Tryptophan Displaying Nanodevices on Macrophage Activation for Preventing Acute Lung Injury

**DOI:** 10.3389/fimmu.2021.750128

**Published:** 2021-09-30

**Authors:** Liya Sun, Rui Wang, Chenchen Wu, Jiameng Gong, Huiqiang Ma, Shan-Yu Fung, Hong Yang

**Affiliations:** ^1^ School of Biomedical Engineering and The Province and Ministry Co-Sponsored Collaborative Innovation Center for Medical Epigenetics, Tianjin Medical University, Tianjin, China; ^2^ Department of Pharmacology, School of Basic Medical Sciences, Tianjin Medical University, Tianjin, China; ^3^ Key Laboratory of Immune Microenvironment and Disease of the Ministry of Education and Department of Immunology, School of Basic Medical Sciences, Tianjin Medical University, Tianjin, China

**Keywords:** immunomodulatory nanoparticles, acute lung injury, Toll-like receptor, peptide, gold nanoparticle, trained immunity

## Abstract

Macrophages play an important role in the initiation, progression and resolution of inflammation in many human diseases. Effective regulation of their activation and immune responses could be a promising therapeutic strategy to manage various inflammatory conditions. Nanodevices that naturally target macrophages are ideal agents to regulate immune responses of macrophages. Here we described a special tryptophan (Trp)-containing hexapeptide-coated gold nanoparticle hybrid, PW, which had unique immunomodulatory activities on macrophages. The Trp residues enabled PW higher affinity to cell membranes, and contributed to inducing mild pro-inflammatory responses of NF-κB/AP-1 activation. However, in the presence of TLR stimuli, PW exhibited potent anti-inflammatory activities through inhibiting multiple TLR signaling pathways. Mechanistically, PW was internalized primarily through micropinocytosis pathway into macrophages and attenuated the endosomal acidification process, and hence preferentially affected the endosomal TLR signaling. Interestingly, PW could induce the expression of the TLR negative regulator IRAK-M, which may also contribute to the observed TLR inhibitory activities. In two acute lung injury (ALI) mouse models, PW could effectively ameliorate lung inflammation and protect lung from injuries. This work demonstrated that nanodevices with thoughtful design could serve as novel immunomodulatory agents to manage the dysregulated inflammatory responses for treating many chronic and acute inflammatory conditions, such as ALI.

## Introduction

Understanding the interactions between nanodevices and the immune system is pivotal to guide nanomaterial design for better biomedical application outcome. When the nanodevices are introduced into the body, they will inevitably encounter the immune system, and may trigger the innate and adaptive immune responses for good or bad. In the field of nanomedicine, majority of the efforts have been focusing on designing “inert” or “invisible” nanodevices to escape elimination by the immune system to achieve prolonged circulation and less immunogenicity. For example, polyethylene glycol (PEG) has been widely used to modify the nanodrug carriers to avoid serum protein adsorption and reduce phagocytosis by immune cells (1–3); CD47 protein is introduced on the surface of the nanodevices to serve as the “don’t eat me” signal (4, 5). However, with increasing understanding of the roles of specific immune cells in the pathophysiological process of human diseases, the phagocytotic properties of innate immune cells to grasp nanodevices could be conversely advantageous for modulation of immune reactions, opening new avenues to treat inflammatory disorders that currently lack of an effective cure.

In contrast to the “immune inert” purpose, the use of nanodevices to modulate innate and adaptive immune responses has sparked significant interests in recent years. For instance, studies have shown that the internalization of the Syk inhibitor (piceatannol)-loaded albumin nanoparticles into neutrophils can block the pro-inflammatory responses of activated neutrophils, serving as a new treatment for diseases associated with exaggerated neutrophil activation (6). A PEGylated polyphenol-based antioxidant, rosmarinic acid (RA), was designed to form nanoparticles (RANPs) to preferentially target the inflamed colon in colitis mice, and significantly reduce colon inflammation by scavenging the reactive oxygen species (ROS) (7). On the other hand, nucleic acid-based Toll-like receptor (TLR) ligands were encapsulated into liposomes to serve as potent adjuvants to boost anti-tumor immune responses (8). Interestingly, “drug-free” nanoparticles (i.e., do not carry any therapeutic agent) have been newly discovered with potent immune modulatory activities. A luminol-conjugated β-cyclodextrin based nanoparticle (LCD NP) was synthesized to effectively inhibit the inflammatory responses, oxidative stress and migration of neutrophils and macrophages to treat acute and chronic inflammatory disorders (9). These findings are encouraging that with proper design, nanoparticles could regulate immune responses, aiding to new immunotherapies to treat various diseases.

Previously, we discovered a “drug-free” peptide-gold nanoparticle (GNP) hybrid, P12, with novel anti-inflammatory activities (10–12). P12 was made of hexapeptides (CLPFFD) wrapping around the surface of a 13-nm GNP. It effectively inhibited multiple TLR signaling pathways in THP-1-derived macrophages and human peripheral blood mononuclear cells (PBMC), and exhibited potent anti-inflammatory activity in a lipopolysaccharide (LPS)-induced acute lung injury (ALI) mouse model (13). This unique bioactivity of P12 was attributed to its surface property associated with the amino acid characteristics in the modifying peptides (10).

Among the 20 natural amino acids, tryptophan (Trp, W) is one of the abundant amino acid residues in the membrane proteins, preferentially in the transmembrane region, suggesting its membrane anchoring property (14). Such a membrane anchoring process involves different interactions between the Trp residues and the lipid bilayer of the membrane. These interactions include the H-bonding, cation-π interaction, and hydrophobic interaction between Trp and the lipid phosphatidylcholine (PC). Thus, the use of Trp-containing peptides modifying the GNP surface may provide attractive properties aiding to the regulation of TLR signaling *in vitro* and *in vivo*.

In this study, we formulated the Trp-containing peptide (CLPWWD) coated GNP hybrid (designated as PW) and investigated its anti-inflammatory activity as well as the possible mechanism(s) of actions *in vitro* and *in vivo*. First, the inhibitory effects of PW on various TLR signaling pathways were confirmed. The global transcriptomic analysis was then applied to obtain the profile of gene expression altered by PW on the regulation of inflammatory responses with/without LPS stimulation. Mechanistically, the inhibitory activity of PW was associated with its endosomal pH modulatory activity. In addition, the up-regulated inhibitory signaling of IRAK-M by PW treatment may also contribute to its novel anti-inflammatory activities. Lastly, the *in vivo* therapeutic efficacy of PW was assessed using two ALI mouse models. This research provides knowledge of understanding the interactions between bioactive nanoparticles with the innate immune system *in vitro* and *in vivo*. It also helps develop immune modulatory nanodevices as a new generation of anti-inflammatory nanomedicine for human inflammatory diseases.

## Materials and Methods

### Materials

Gold(III) chloride trihydrate (99.9%), phorbol myristate acetate (PMA), LPS (E-coli O111:B4, for mice), chloroquine (CHQ), wortmannin, fucoidan, mannan, genistein, methyl-β-cyclodextrin (MβCD), chlorpromazine (CPZ) and SP600125 were purchased from Sigma (Sant-Louis, MO, USA). Cytochalasin D (CytoD) and filipin III (filipin) were purchased from Cayman (Ann Arbor, MI, USA), while Nystatin was from MCE (Monmouth, NJ, USA). Peptides were synthesized from Nanjing Jietai Biological Company (Nanjing, China). The human monocytic THP-1 cell line was obtained from ATCC (Rockefeller, MD, USA). THP-1 reporter cell lines (XB and ISG), LPS-EK (LPS from E. coli K12, for cells), Poly I/C (high molecular weight, HMW), resiquimod (R848), Pam3CSK4, Zeocin and QUANTI-Blue™ solution were purchased from InvivoGen (San Diego, CA, USA). RPMI 1640 medium, phosphate buffered saline (PBS) and fetal bovine serum (FBS) were purchased from Biological Industries (Kibbutz Beit Haemek, Israel). L-glutamine and sodium pyruvate were from Gibco (Grand Island, NY, USA). MTS assay was purchased from Promega (Madison, WI, USA). Human ELISA kits (MCP-1, TNF-α and IL-6) and mouse IL-10 ELISA kits were purchased from Invitrogen (Grand Island, NY, USA). Human ELISA kit IL-12/IL-23 p40 was purchased from R&D Systems (Minneapolis, MN, USA). Tris buffered saline (TBS), Liu stain and red blood cell (RBC) lysis buffer were purchased from Solarbio (Beijing, China). The primary antibodies against phosphorylated p65 (#3033S), IRF3 (#4947S) and STAT1 (#9167S), IκBα (#9242S), β-actin (#8457S), IRF7 (#13014S) and IRAK-M (#4369S) as well as the HRP conjugated anti-rabbit (#7074S) antibodies were purchased from Cell Signaling Technology (Boston, MA, USA). The RIPA lysis buffer, Halt protease and phosphatase inhibitor cocktail, the Coomassie Plus (Pierce) of Bradford assay and pHrodo red-labeled 10,000 MW dextran were from Thermo Fisher Scientific (Waltham, MA, USA). Bovine serum albumin (BSA) was purchased from Genview (Houston, TX, USA). Tween 20, sodium citrate tribasic dihydrate and cholesterol were purchased from Sangon Biotech (Shanghai, China). Soy Lecithin was obtained from Tywei (Shanghai, China). RNeasy Plus Mini kit for total RNA extraction was obtained from Qiagen (Hilden, Germany). Cy5-PEG5000-SH was obtained from Ponsurebio (Shanghai, China). 3,3′-dioctadecyloxacarbocyanine perchlorate (DiO) and 2-(4-Amidinophenyl)-6-indolecarbamidine dihydrochloride (DAPI) were purchased from Beyotime (Shanghai, China).

### Methods

#### Fabrication of Peptide-GNP Hybrids

Bare gold nanoparticles (GNPs) were synthesized based on the literature and our previous work (10, 15). The pepide-GNP hybrids were prepared by mixing 1 volume of different peptide solutions (CLPWWD, CLPFFD, CLPLLD, CLPIID, CLPAAD, CLPSSD and CLPTTD at a concentration of 1 mM) with 10 volumes of bare GNPs and kept in dark for at least 24 h. For fluorescent nanoparticles (PW-Cy5), one volume of peptide stock solution (1 mM) containing 1% Cy5-PEG5000-SH (molar ratio) was mixed with ten volumes of synthesized GNP solution. The peptide-GNP solution was filtered through a syringe filter (0.22 µm, Millipore, Billerica, MA, USA) and washed three times with sterile PBS to remove unbound peptide ligands by centrifugation (14,000 rpm for 30 minutes at 4°C). The peptide GNPs were resuspended in PBS or cell culture medium at the desired concentrations prior to the cell culture experiments or animal studies.

#### Liposome Fabrication

Liposomes were prepared by the method of thin film hydration and then purified by centrifugation (14000 rpm, 1 h, 4°C). Briefly, the soy lecithin (180 mg) and cholesterol (60 mg) were dissolved in 20 mL of the chloroform-methanol mixture (3:1, v/v). The thin lipid film was formed by rotary evaporation at 37°C. The lipids were re-hydrated in PBS (15 mL), and the suspension was dispersed by ultrasound, followed by filtering through a 0.22 µm microporous membrane (Millipore, Billerica, MA, USA). The liposomes were stored at 4°C prior to use.

#### Characterization of Peptide-GNP Hybrids and Their Interaction With Liposomes

The morphology of the hybrid PW and the mixtures of liposomes with PW or PT were imaged on a transmission electron microscope (TEM) (HT7700, Hitachi, Tokyo, Japan) with an accelerating voltage of 80 kV. The hydrodynamic diameter and Zeta potential of the bare GNPs and PW were determined by using the Zetasizer instrument (Nano-ZS, Malvern, Worcestershire, UK).

#### Cell Culture and Treatments

The THP-1 cells were cultured in the complete RPMI 1640 medium containing 10% FBS, 2 mM L-glutamine and 1 mM sodium pyruvate with 5% CO_2_ at 37°C. The complete culture medium was supplemented with Zeocin (200 or 100 μg/mL) as the selection medium for THP-1-XBlue cells or THP-1-ISG cells, respectively. These cells were seeded into culture plates with the addition of PMA (50 ng/mL) for 24 h to differentiate into macrophages; they were then washed twice with PBS and rested for 48 h prior to further experiments.

The seeded macrophages were treated with the hybrids, different TLR ligands (LPS, Pam3CSK4 and poly I/C) or both the hybrid and TLR stimulus for various time periods; the culture medium and the cells lysates were collected for further analysis. For R848 stimulation, THP-1 monocytes were seeded in a cell culture plate overnight and then treated with PW and R848 (10 μg/mL) for 24 h before further analysis.

#### Cell Viability Test

The derived macrophages (1×10^5^ cells/well) were seeded into a 96-well plate and treated with PW for 24 h. MTS reagent (20 µL/well) was directly added to each well and incubated for 1-2 h. The absorbance at 490 nm was recorded on a microplate reader (TECAN, Mannedorf, Zurich, Switzerland). The percentage of viable cells was estimated in comparison to the untreated group as 100%.

#### Reporter Cell Assay by QUANTI-Blue

The derived macrophages or THP-1 monocytes (1×10^5^ cells/well) were seeded in a 96-well flat- or U-bottom plate, respectively. After treatments, the culture media were collected and centrifuged to remove the hybrids. The supernatants (20 µL) were transferred into a new 96-well plate and mixed with the QUANTI-Blue solution (180 µL) and incubated at 37°C for 1-2 h for the color development. The color change was quantified by measuring the absorbance at 655 nm on a microplate reader (TECAN, Mannedorf, Zurich, Switzerland) to examine NF-κB/AP-1 or IRF activation.

#### Immunoblotting Analysis

THP-1 monocytes (1×10^6^ cells/well) were seeded into a 12-well plate and differentiated into macrophages. After various treatments, cells were lysed in ice-cold RIPA lysis buffer. The total protein concentration of the lysates was quantified and adjusted prior to the protein separation by 10% SDS-PAGE. The separated proteins were transferred to PVDF membranes (Immobilon-P, Millipore, Billerica, MA, USA). The membranes were blocked and blotted with primary antibodies against β-actin, IκBα, p-p65, p-IRF3, p-STAT1, IRF7 and IRAK-M at 4°C overnight. They were then blotted with HRP labelled secondary antibody for 1 h at room temperature, and imaged by the chemiluminescence method (ECL, Millipore, Billerica, MA, USA) on a ChemiDoc MP imaging system (Bio-Rad, Hercules, CA, USA). The protein band densitometry was analyzed using ImageJ software (NIH, Bethesda, MD, USA).

#### Cytokine Analysis

THP-1 monocytes (5×10^5^ cells/well) were seeded into a 24-well plate and differentiated into macrophages. After various treatments for 24 h, the culture medium was centrifuged (14000 rpm, 4°C, 30 min), and the supernatant was collected for the analysis of different cytokines by ELISA following the manufacturer’s instructions.

#### Confocal Fluorescence Imaging

THP-1 cells (2.4×10^5^ cells/well) were seeded in a 20 mm glass bottom dish (NEST, Wuxi, China) and differentiated into macrophages. To assess the endosomal acidification, cells were incubated with pHrodo red-labeled dextran (10,000 MW, 10 µg/mL) and PW (200, 100 and 50 nM) or chloroquine (30 µM) for 5 h and processed for confocal imaging. To examine the uptake of PW in macrophages, cells were incubated with Cy5-labelled PW (PW-Cy5, 10 nM) for 5 h, and the cell membrane and nucleus were then stained with DiO and DAPI, respectively. Cells were washed with PBS and imaged on a confocal microscope (LSM900, Leica Microsystems Inc., Wetzlar, Hessen, Germany). The fluorescence of pHrodo red (ex: 565 nm; em: 585 nm) and Cy5 (ex: 640 nm; em: 670 nm) in the cells was quantified by the ImageJ software. For each condition, at least 30 cells were quantified with three independent repeats.

#### Cellular Uptake Analysis

To analyze the endocytotic pathways of entry of PW, THP-1-derived macrophages were pre-treated with various endocytotic inhibitors 30 min prior to the addition of the PW-Cy5 (10 nM) for 5 h. These inhibitors included CytoD (3 μM) and wortmannin (10 μM) for macropinocytosis, fucoidan (25 μg/mL) for scavenger receptor-mediated endocytosis, mannan (500 μg/mL) for mannose receptor-mediated endocytosis, genistein (200 μM), nystatin (10 μM), MβCD (5 mM) and filipin (10 μM) for caveolae/lipid raft-mediated endocytosis, and CPZ (10 μM) for clathrin-mediated endocytosis. After treatments, cells were collected, washed and fixed by 4% paraformaldehyde for 15 min. They were washed and resuspended in 0.9% NaCl solution containing 0.1 M NaOH (to remove PW-Cy5 adsorbed on the membrane surface). After final wash step, cells were resuspended in PBS for flow cytometric analysis.

#### RNA-Seq Analysis

THP-1 monocytes (2×10^6^ cells/well) were seeded into a 12-well plate and differentiated into macrophages. After various treatments (Unstim, PW, LPS and LPS-PW) for 4 h, the total RNA was extracted using RNeasy mini kit and assessed by a Nanodrop Lite Spectrophotometer (Thermo Scientific™, Waltham, MA, USA) for the purity. The RNA-Seq was done using Illumina Novaseq 6000 platform by Novogene Co., LTD (Beijing, China).

Differential expression analysis was performed using the DESeq2 R package (1.26.0). Genes with the criteria of an adjusted p-value < 0.05, |log2 (fold change)| > 0.5 or 2 were assigned as differentially expressed. The pheatmap R package (1.0.12) was used to produce heatmaps of differentially expressed genes (DEGs) between groups: PW vs. Unstim and LPS-PW vs. LPS. The ggplot2 R package (3.3.5) was used to generate volcano plots of DEGs.

ClusterProfiler R package (3.14.3) was used to perform the KEGG pathway enrichment analysis. KEGG pathways with adjusted p-values < 0.05 were considered statistical significance in the enrichment analysis. The most enriched 20 KEGG pathways were shown in the bar graph. ClueGO (2.5.8), a plug-in of Cytoscape, was used to show the enriched DEGs in the most significant KEGG pathways with a p value < 0.05 as the cut-off criterion.

#### Acute Lung Injury Murine Model

C57BL/6 wild-type male mice (6-8 weeks from SPF Biotechnology Co., Ltd, Beijing, China) were used to replicate the ALI mouse model by intratracheal LPS administration. All the surgical procedure was done under the 1% sodium pentobarbital anesthesia (45 mg/kg) through intraperitoneal injection. PW (1.25 nmol/kg) was given through intratracheal injection 2 h before LPS (10 mg/kg) challenge. Mice were sacrificed 24 h after LPS challenge for the analyses of lung inflammation and injury. All mouse studies were performed according to the guidelines of the Institutional Animal Care and Use Committee of Tianjin Medical University.

#### BALF Collection and Differential Cell Counting

At the end of the ALI model, mice were under tracheotomy, and ice-cold sterile PBS (0.8 mL) was injected through the trachea to the lung twice. The bronchoalveolar lavage fluid (BALF) was collected 30 s after injection, and centrifuged at 1000 rpm for 10 min at 4°C. The supernatants were stored at −80°C for cytokine analysis. The cell pellets were processed with 4% RBC lysis buffer and resuspended in PBS for total cell counting on a hemocytometer. Aliquots of the cell suspensions were cytospined onto a glass slide, stained with Liu stain, and imaged on an up-right microscope (ECLIPSE Ni-U, Nikon, Tokyo, Japan) for differential cell counting. A total of at least 300 cells were counted for each sample.

#### Lung Injury Score and W/D Ratio

In a different set of experiment, the left lung was collected, fixed in 4% paraformaldehyde, dehydrated and embedded in paraffin for histopathological sectioning. The tissue sections were stained with hematoxylin and eosin (H&E) and imaged on an up-right microscope (ECLIPSE Ni-U, Nikon, Tokyo, Japan). For each sample, at least 20 images at 400x amplification were blindly scored by three independent researchers on the five histopathological features: alveolar neutrophils, interstitial neutrophils, hyaline membranes, proteinaceous debris, and alveolar septal thickening (16). The remaining lungs were processed for the analysis of W/D ratio as follows. The fresh lung tissues were first weighed, and wrapped in aluminum foil for drying in an oven at 60°C for 48 h. They were weighed again, and the weight ratio was calculated to obtain the W/D ratio.

#### Statistical Analysis

All statistical analysis was carried out using GraphPad Prism 7.0. All data are expressed as means ± SEM, and a p-value of < 0.05 was considered statistically significant. For comparison between two groups, the student t-test was used, whereas one way ANOVA with Bonferroni correction was performed for multiple comparison among groups.

## Results

### Synthesis and Characterization of the Peptide-GNP Hybrid PW

**Figure 1:**
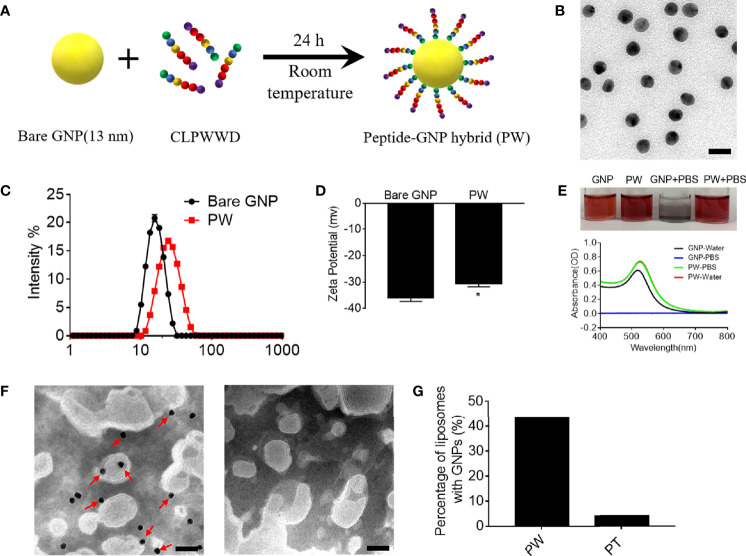
Preparation of the peptide-GNP hybrid PW and its physicochemical characterization. **(A)** A schematic diagram of the synthesis of the peptide-GNP hybrid PW. **(B)** The TEM image of PW; scale bar = 20 nm. **(C)** The size distribution of the bare GNP and PW. **(D)** The zeta-potential of the bare GNP and PW. **(E)** The photograph (top) and UV-Vis spectra (bottom) showing the stability of bare GNP and PW in water and PBS. **(F)** The TEM images of the nano-hybrids PW (left) and PT (right, coated with the peptide CLPTTD) with liposomes showing that PW tends to interact with liposomes, but the control nano-hybrid PT does not; scale bar = 50 nm, PW, PT = 5 nM, phospholipid = 1.67 mg/mL. **(G)** The percentage of liposomes interacting with the nanohybrids; over 200 liposomal vesicles were counted. N ≥ 3, *p < 0.05.

We previously developed a novel anti-inflammatory peptide-GNP hybrid (P12) that can inhibit multiple TLR signaling pathways without carrying any drug compound (10). P12 was made of a hexapeptide (CLPFFD) shell and a 13-nm GNP core. We demonstrated that the FF region in the peptide sequence plays an important role for TLR inhibition, and the hydrophobicity of this region contributes to the potency of such inhibitory activity (11). Among 20 natural amino acids, tryptophan (W) has a hydrophobic side chain and preferentially interacts with cell membrane; hence, it would be very interesting to know whether the replacement of FF with WW could render different immunomodulatory activities of P12. Accordingly, the CLPWWD hexapeptide was used to coat the GNP to obtain a new hybrid designated as PW (**Figure 1A**). The size and morphology of the PW were characterized by transmission electron microscopy (TEM). As shown in **Figure 1B**, the PW was monodispersed with a uniform spherical structure; its hydrodynamic diameter was analyzed to range from 11.2 ± 0.5 to 68.6± 5.0 with the peak size of 26.0 nm (PDI: 0.13 ± 0.01) by dynamic light scattering (DLS) (**Figure 1C**), which was 9.4 nm larger than the bare GNP (the peak size of 16.6 nm, range from 7.9 ± 0.3 to 34.4 ± 1.5; PDI: 0.04 ± 0.01), suggesting that the formation of the peptide coating on the GNP surface. The PW had a zeta-potential of −30.6 ± 2.1 mV, slightly less negative than the bare GNP (−36.0 ± 2.4 mV) (**Figure 1D**). However, this quite negative zeta-potential value was essential for the stability of PW in aqueous solution. In fact, PW was stable even in PBS solution while the bare GNP formed aggregates indicated by the solution color as well as the optical absorption at 519 nm (**Figure 1E**). These results suggested that peptide coating on the GNP surface significantly improved the stability of GNPs at the physiological condition.

Based on the optical characteristics of Trp that has a strong absorbance at 280 nm wavelength, we were able to estimate the number of Trp-containing peptide molecules conjugated on the GNP surface. It was found that each PW had approximately 1483 ± 344 peptides coating on the GNP surface.

To examine whether PW preferentially interacts with the cell membrane, we used liposomes as the artificial cell membrane and observed their interactions by TEM. A control hybrid PT, which W was replaced with the non-membrane anchoring amino acid threonine (T), was employed for comparison. As shown in **Figure 1F**, there were more nanoparticles (PW) observed on the liposome membranes (indicated by the red arrows), but none of PT were seen (right panel). These images were further quantified to show that more than 43% of liposomes had PW on the membranes, significantly higher than that (< 4%) for PT (**Figure 1G**). These observations suggested that PW had a stronger membrane affinity due to the membrane anchoring property of the Trp residues.

### The Immunomodulatory Activity of PW to Macrophages

**Figure 2:**
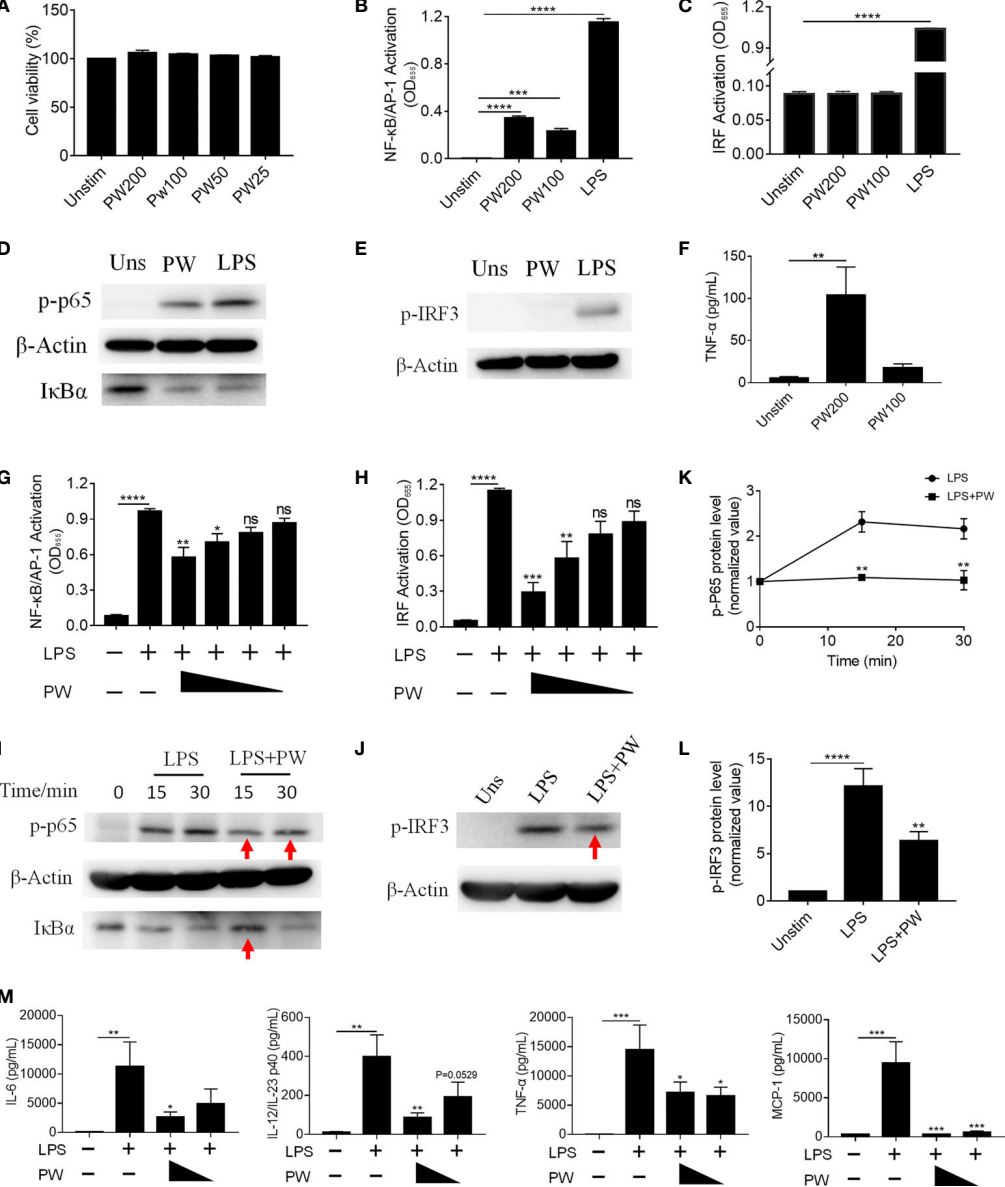
The immunomodulatory activity of PW on TLR4 signaling in THP-1-derived macrophages. **(A)** The effect of the PW treatment on the cell viability measured by the MTS assay; PW concentrations: 200, 100, 50 and 25 nM. **(B, C)** The effect of PW treatment alone on the activation of NF-κB/AP-1 **(B)** and IRF **(C)** in the THP-1 reporter cell-derived macrophages. **(D, E)** Immunoblotting validating the effect of PW (200 nM) on the phosphorylation of p65 (p-p65) and degradation of IκBα at 1 h for NF-κB activation **(D)** and the phosphorylation of IRF3 (p-IRF3) at 1 h for IRF activation **(E)** in THP-1-derived macrophages; β-actin as the internal control. **(F)** TNF-α production induced by PW at 100 and 200 nM. **(G, H)** The inhibition of LPS-induced activation of NF-κB/AP-1 **(G)** and IRF **(H)** by PW with various concentrations (200, 100, 50 and 25 nM) in the THP-1 reporter cell-derived macrophages. **(I, J)** Immunoblotting confirming the inhibitory effect of PW (200 nM) on the LPS-induced phosphorylation of p65 (p-p65) and degradation of IκBα for NF-κB activation **(I)** and the phosphorylation of IRF3 (p-IRF3) at 1 h for IRF activation **(J)**; β-actin as the internal control; the time course of p65 phosphorylation **(K)** and the IRF3 phosphorylation **(L)** were quantified from **(I)** and **(J)**, respectively. **(M)** Inhibition of IL-6, IL-12/IL-23p40, TNF-α and MCP-1 production upon 24 h LPS stimulation by PW (200 and 100 nM) in THP-1-derived macrophages. N ≥ 3, LPS = 10 ng/mL, ns, not significant, *p < 0.05, **p < 0.01, ***p < 0.001, ****p < 0.0001 *vs*. LPS.

Macrophages are important phagocytic immune cells in initiating pro-inflammatory responses for the host defense against pathogens and participating in the pathogenesis of many diseases. To study how PW influences the immune responses of macrophages, we employed the human monocytic THP-1-derived macrophages with/without the reporter system of the transcription factors NF-κB/AP-1 (THP-1 XBlue cells) and IRF (THP-1 Blue ISG cells). First, we confirmed that PW had no effect on the viability of macrophages up to 200 nM (**Figure 2A**). Using the two reporter cells, we found that PW itself could mildly activate NF-κB/AP-1, but much less than the strong TLR4 agonist LPS (10 ng/mL) (**Figure 2B**); such an effect was not observed on the activation of IRF (**Figure 2C**). These observations were further confirmed at the protein level, where NF-κB was activated by PW as indicated by the increase in the phosphorylation of the NF-κB subunit p65 (p-p65) and the degradation of the NF-κB inhibitor unit IκBα, while PW had no effects on the phosphorylation of IRF3 (p-IRF3) for IRF3 activation (**Figures 2D, E**). Furthermore, PW at a higher concentration of 200 nM was able to induce the production of the pro-inflammatory cytokine TNF-α (**Figure 2F**). Together, these results suggested that PW alone could trigger the pro-inflammatory responses of macrophages.

Interestingly, when macrophages were activated by LPS, PW treatment on the contrary inhibited the activation of both NF-κB/AP-1 and IRF in a concentration dependent manner (**Figures 2G, H**). Such inhibition was also verified by immunoblotting (**Figures 2I–L**), where the LPS-induced phosphorylation of p65 and IRF3 as well as the degradation of IκBα were decreased by PW (indicated by red arrows). Moreover, PW was able to reduce the production of many pro-inflammatory cytokines, including IL-6, IL-12/IL-23p40, TNF-α and MCP-1, triggered by LPS stimulation (**Figure 2M**). Surprisingly, neither the peptide nor the GNPs alone could activate NF-κB/AP-1 of resting macrophages or reduce the activation of NF-κB/AP-1 of LPS stimulated macrophages (**Supplementary Figure S1**). Only when the peptide and GNP formed nano-hybrids did they exhibit immunomodulatory activities. These results demonstrated that PW itself seemed to activate macrophages and induce mild pro-inflammatory responses, but under strong inflammatory stimulation by LPS, PW instead exhibited potent anti-inflammatory activity. Such an opposite effect of PW makes it an interesting and unique class of immunomodulatory nanoparticles.

### Preferential Inhibition of Endosomal TLR Signaling Pathways by PW

**Figure 3:**
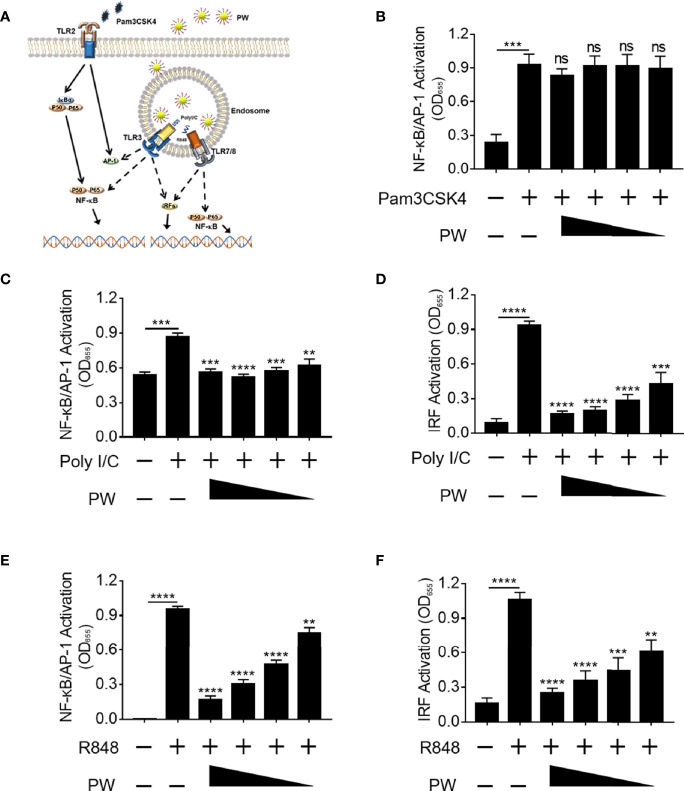
Preferential inhibition of endosomal TLR signaling by PW in THP-1 reporter cells and their derived macrophages. **(A)** Illustration of the effects of PW on the cell surface TLR2 and the endosomal TLRs 3 and 7/8 signaling. **(B)** PW could not inhibit NF-κB/AP-1 activation induced by the TLR2 ligand Pam3CSK4 (10 ng/mL) in THP-1 reporter cell-derived macrophages. The inhibition of NF-κB/AP-1 **(C)** and IRF **(D)** by PW in TLR3 signaling stimulated by the Poly I/C (50 μg/mL) in THP-1 reporter cell-derived macrophages. The inhibitory effect of PW on the activation of NF-κB/AP-1 **(E)** and IRF **(F)** induced by the TLR7/8 ligand R848 (10 µg/mL) in THP-1 reporter cells. The concentrations of PW used: 200, 100, 50 and 25 nM; N ≥ 3, ns, not significant, **p < 0.01, ***p < 0.001, ****p < 0.0001.

Next, we would like to address whether the inhibitory activity of PW was specific to TLR4. Among all known TLRs, we investigated the effects of PW on the cell surface TLR2 and the endosomal TLRs 3 and 7/8 (**Figure 3A**). We found that PW had no effects on the activation of NF-κB/AP-1 stimulated by the TLR1/2 ligand Pam3CSK4 (**Figure 3B**). However, PW treatment significantly reduced the activation of NF-κB/AP-1 and IRF triggered by the TLR3 ligand Poly I/C as well as the TLR7/8 ligand R848 in a concentration-dependent fashion (**Figures 3C–F**). In addition, PW was able to inhibit the secretion of IL-6, TNF-α and MCP-1 upon R848 stimulation (**Supplementary Figure S2**). These results indicated that PW had a relatively broad inhibitory activity preferentially on the endosomal TLR pathways.

### Transcriptomic Analysis by RNA-Seq to Define the Immunomodulatory Activities of PW on Macrophages

**Figure 4:**
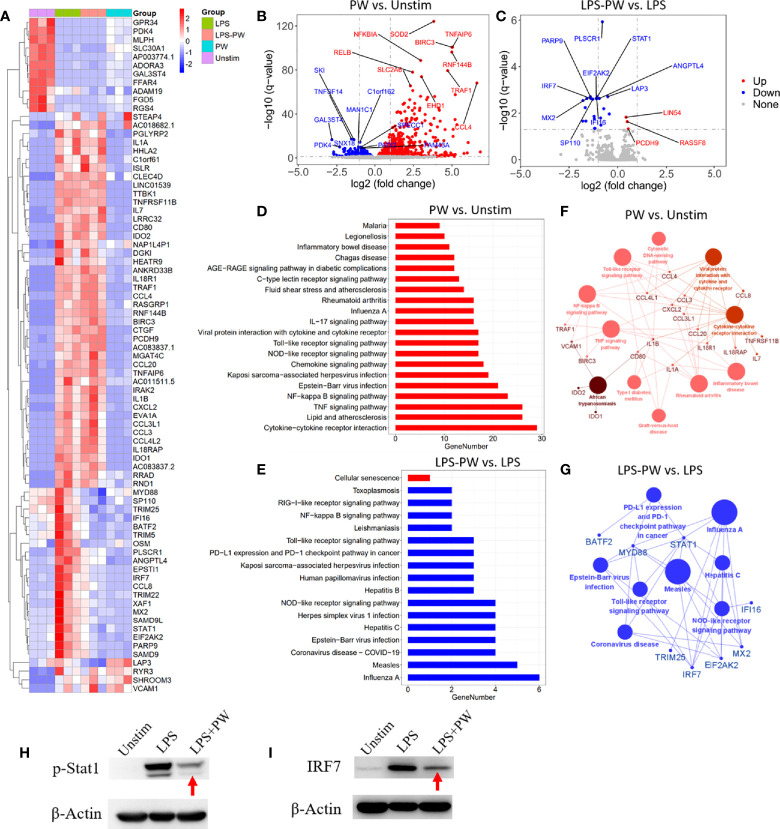
Transcriptome analysis by RNA-Seq defining the immunomodulatory activities of PW in THP-1-derived macrophages. **(A)** The heatmap of the top 79 differentially expressed genes (DEGs) among the four experimental groups: LPS stimulation (LPS), LPS with PW treatment (LPS-PW), PW treatment only (PW) and unstimulated control (Unstim); each with three biological replicates; top 57 DEGs of adjusted p-value < 0.05 and |log2 (fold change)| > 2 were identified for PW vs. Unstim group, and 22 DEGs with adjusted p-value < 0.05 and |log2 (fold change)| > 0.5 were shown for LPS-PW vs. LPS group. **(B, C)** Volcano plots showing all the gene expression pattern and highlighting the top 10 up- or down-regulated DEGs in PW vs. Unstim group **(B)** and LPS-PW vs. LPS group **(C)**; the red, blue and gray color represents up-regulated, down-regulated and non-significant genes, respectively; the threshold line setting for adjusted p-value = 0.05 and |log2 (fold change)| = 1. **(D, E)** KEGG pathway enrichment analysis for PW vs. Unstim **(D)** and LPS-PW vs. LPS **(E)**; all the pathways were significantly enriched with an adjusted p-value < 0.05; the red and blue color represents up-regulated and down-regulated pathways, respectively. **(F G)** Interaction analysis of pathways and related genes by ClueGO for PW vs. Unstim group **(F)** and LPS-PW vs. LPS group **(G)**; the red and blue color represents up-regulated and down-regulated pathways and genes, respectively; the cut-off p-value = 0.05. **(H, I)** Immunoblotting confirming the inhibition of STAT1 activation (phosphorylation of STAT1, p-STAT1) at 2h **(H)** and IRF7 expression at 24 h **(I)** by PW under LPS stimulation; β-actin as the internal control. PW = 100 nM, LPS = 10 ng/mL, N ≥ 3.

To better define the pro-inflammatory and anti-inflammatory activity of PW, the high-throughput RNA-Seq analysis was conducted to obtain the transcriptomic profiles of PW treatment on the macrophages in the absence and presence of the LPS stimulation. The differential gene expression profiles of PW were visualized on the heat map (**Figure 4A**), where top 79 differentially expressed genes (DEGs) were displayed. PW induced 57 genes with > 2 folds of expression changes in comparison with the unstimulated control group. Interestingly, under LPS stimulation, PW was able to decrease 19 LPS up-regulated genes to the level similar to the unstimulated control group. The volcano plots showed all the gene expression pattern and highlighted the top 10 up- or down-regulated DEGs (**Figures 4B, C**). Collectively, the transcriptome analysis showed that the PW alone had mild pro-inflammatory effects, but exhibited significant anti-inflammatory activity in the presence of LPS stimulation.

The KEGG pathway enrichment analysis identified 20 and 17 significantly enriched pathways in the PW vs. Unstim group and the LPS-PW *vs*. LPS group, respectively (**Figures 4D, E**). PW treatment alone up-regulated the immune-related signaling pathways, such as Toll-like receptor signaling pathways, NF-κB signaling pathways, and viral infection-related signaling pathways (**Figure 4D**). However, with the LPS stimulation, PW in contrast down-regulated those immune-related signaling pathways including Toll-like receptor signaling pathways and NF-κB signaling pathways (**Figure 4E**). These up- and down-regulated pathways and essential genes were plotted into the pathway networks as shown in **Figures 4F, G**. In the networks, many cytokine genes were found to be elevated in PW vs. Unstim group, while the STAT1 and IRF7 transcription factors as well as the primary adaptor protein in TLR pathway MyD88 were down regulated in the LPS-PW vs. LPS group. The changes in the phosphorylation of STAT1 and IRF7 expression were confirmed by immunoblotting (**Figures 4H, I**). The reporter assay, cytokine measurements and the transcriptomic profiles coherently revealed that PW had opposite immunomodulatory effects depending on the presence of the TLR stimuli.

### The Mechanism(s) of Actions of the Novel Immunomodulatory Activities of PW

**Figure 5:**
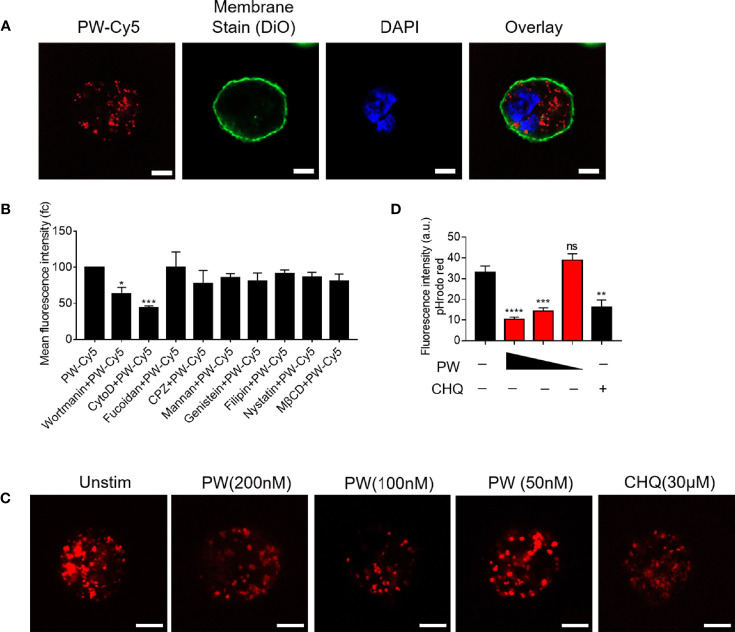
The uptake of PW in THP-1-derived macrophages and the modulation of the endosomal pH. **(A)** Confocal images of macrophages treated with Cy5 labeled PW (PW-Cy5, red) for 5 h with the cell membrane stained with DiO (green) and the nucleus stained with DAPI (blue). **(B)** The quantitative analysis of PW-Cy5 in the macrophages with the pre-treatment of various endocytic pathway inhibitors for 5 h in comparison with the no inhibitor control by flow cytometry; N ≥ 3. **(C)** Confocal images of macrophages treated with PW (200, 100 and 50 nM) and the well-known pH modulator chloroquine (CHQ, 30 μM); cells were simultaneously treated with the endosomal pH indicator pHrodo red dextran (10 μg/mL, red); scale bar = 5 μm. **(D)** Quantitative analysis of the pHrodo red fluorescence signals as an indicator of endosomal pH; over 30 cells were quantified from three independent experiments; the fluorescence intensity is reversely proportional to the pH. *p < 0.05, **p < 0.01, ***p < 0.001, ****p < 0.0001; ns, not significant.

We previously found that the inhibitory activity of the peptide-GNP hybrid P12 on TLR signaling was through the modulation of the endosomal pH (11). We expected that PW may have a similar mechanism as well. The uptake experiment by the confocal microscopy indeed showed that PW was internalized by macrophages (**Figure 5A**). To elucidate which internalization pathways were involved in the PW uptake, different chemical inhibitors were used to specifically inhibit the uptake pathway. It was found that the micropinocytosis inhibitors wortmannin and CytoD could significantly reduce the uptake of PW (**Figure 5B**), suggesting that PW was internalized through the micropinocytosis. As expected, the internalized PW at higher concentrations (100 and 200 nM) could block the endosomal acidification probed by the endosomal pH indicator (pHrodo red), where the dimmer fluorescence signals indicated a higher endosomal pH; the same phenomenon was seen with the well-known pH modulator chloroquine (CHQ) serving as a positive control (**Figures 5C, D**). These results suggested that the endosomal pH modulation contributed to the observed concentration dependent inhibition of TLR signaling by PW.

**Figure 6:**
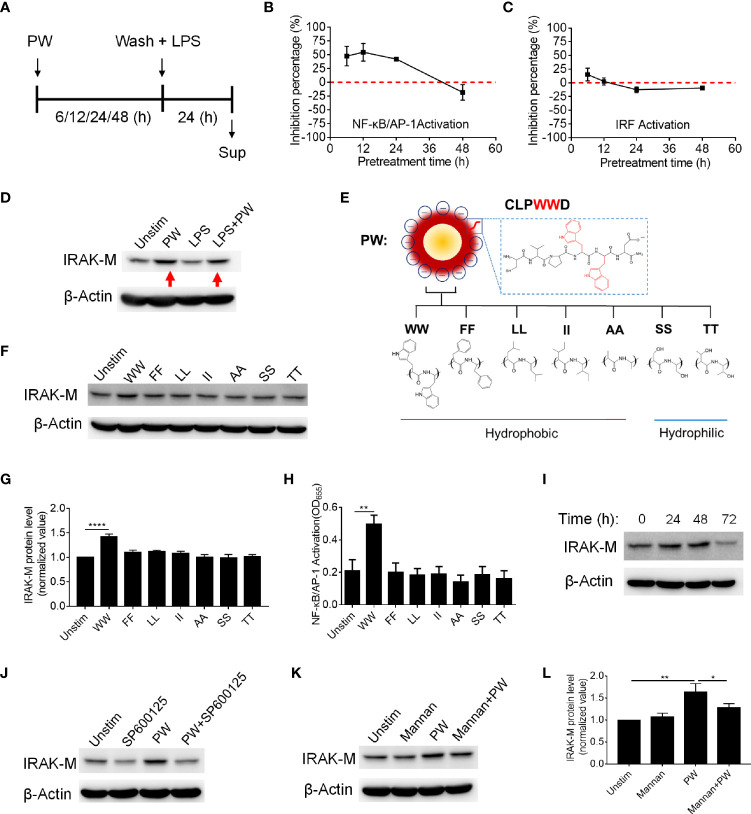
The priming effects of PW on the TLR4 inhibition in relationship with the tryptophan dependent expression of IRAK-M in THP-1-derived macrophages. **(A)** The scheme of PW priming (100 nM) for different time periods (6, 12, 24 and 48 h) followed by LPS stimulation (10 ng/mL) for 24 h. **(B, C)** The time effect of PW pre-treatment on its inhibitory capacity on the activation of NF-κB/AP-1 **(B)** and IRF **(C)** upon LPS stimulation. **(D)** Immunoblotting showing the IRAK-M expression at 24 h induced by PW (100 nM) in the absence and presence of LPS (10 ng/mL) stimulation; β-actin as the internal control. **(E)** A scheme showing the mutation of two adjacent tryptophan residues (WW) in the peptide coating of PW to other two hydrophobic amino acids: phenylalanine (FF), leucine (LL), isoleucine (II) and alanine (AA) or hydrophilic amino acids: serine (SS) and threonine (TT). **(F)** The effect of the mutated hybrids (100 nM) displaying different amino acid residues on the IRAK-M expression at 24 h by immunoblotting; β-actin as the internal control. **(G)** Quantification of the IRAK-M expression in **(F)**. **(H)** The effect of the mutated hybrids (100 nM) on the NF-κB/AP-1 activation in the THP-1 reporter cell-derived macrophages. **(I)** The time course of IRAK-M expression induced by PW (100 nM); β-actin as the internal control. **(J)** Immunoblotting showing the IRAK-M expression at 24 h induced by PW (100 nM) in the absence and presence of SP600125 (10 µM); β-actin as the internal control. **(K)** Immunoblotting showing the IRAK-M expression at 24 h induced by PW (100 nM) with/without the pretreatment of mannan (5 µg/mL) for 0.5 h; β-actin as the internal control. **(L)** Quantification of the IRAK-M expression in **(K)**. N ≥ 3, *p < 0.05, **p < 0.01, ****p < 0.0001.

In addition to endosomal pH modulation, there may be other mechanisms of action of PW due to its unique opposite immunomodulatory activity. Based on the above discovery (**Figures 2**, **4**), we hypothesized that the inhibitory effects of PW on the TLR signaling may be primed by the PW treatment, in analogy to the LPS tolerance effects (17). To test this hypothesis, we pre-treated the reporter cell-derived macrophages with PW (100 nM) for different time periods (6, 12, 24 and 48 h), followed by the removal of PW and stimulation with LPS for 24 h to assess the activation of NF-κB/AP-1 and IRF (**Figure 6A**). Interestingly, we found that the PW pre-treatment (for up to 24 h) could inhibit the LPS-induced NF-κB/AP-1 activation; the inhibitory effects diminished and disappeared with longer pre-treatment (48 h) (**Figure 6B**). However, PW pre-treatment appeared to have shorter (6 h) and less priming effects on inhibition of the LPS-induced IRF activation (**Figure 6C**). These results revealed that although PW alone could trigger some inflammatory responses in the macrophages, such reaction may also activate certain regulatory signaling that could last for up to 48 h of priming for the second strike of strong inflammatory signals.

There are many negative regulators reported in controlling the TLR signaling cascades driving NF-κB activation, including SOCS1/3, A20, CYLD and SHP1 (18, 19). Among them, interleukin-1 receptor-associated kinase 3 (IRAK3 or IRAK-M) is a well-known one inhibiting TLR signaling, especially present in the monocytes and macrophages (20). It has been reported to participate in the signaling establishing LPS tolerance of macrophages (21). Thus, we speculated that IRAK-M may be involved in the inhibitory mechanisms of PW on TLR signaling. Interestingly, we found that PW could significantly elevate the expression of IRAK-M in the absence or presence of LPS stimulation (**Figure 6D**). The up-regulation of IRAK-M by PW was specific to the presence of WW region in the peptide sequence, as replacing WW with FF, LL, II, AA, SS, or TT (**Figure 6E**) could not induce the expression of IRAK-M (**Figures 6F, G**). This WW specific induction of IRAK-M expression was correlated well with the NF-κB activation by PW (**Figure 6H**). We also examined the time course of IRAK-M expression upon PW treatment and found that the IRAK-M expression increased with time and reached the maximum expression at 48 h, but significantly deceased at 72 h (**Figure 6I**). This time course of IRAK-M expression, to our surprise, had nice correlation with the observed priming effects of PW on inhibiting LPS-induced NF-κB activation (**Figure 6B**), where no inhibitory effect was seen at the condition of 48 h pre-treatment of PW with 24 h stimulation of LPS, at which the IRAK-M expression significantly dropped 72 h after PW treatment. These observations indicated that the inhibition of NF-κB by PW treatment might be also associated with the up-regulation of IRAK-M, while the inhibition of IRF by PW was primarily governed by the endosomal pH modulation.

Next, we aimed to address how PW induced IRAK-M expression. It was found that IRAK-M expression is regulated by JNK activation (22). When the macrophages were treated with the JNK inhibitor (SP600125), the IRAK-M expression induced by PW was down-regulated (**Figure 6J** and **Supplementary Figure S3**), confirming that JNK activation participated in the IRAK-M expression. To further explore how the PW-induced IRAK-M expression was triggered at the cell surface, we examined the mannose receptor as it has been reported to be utilized by a Trp containing protein to induce IRAK-M expression in macrophages (23, 24). We pretreated the THP-1-derived macrophages with an optimized concentration of the mannose receptor ligand mannan (**Supplementary Figure S4**) to block the interaction of PW with the receptor without activating it. Interestingly, the up-regulation of IRAK-M by PW was significantly reduced (**Figures 6K, L**) with the pretreatment of mannan at a low concentration (5 µg/mL), suggesting that the mannose receptor was involved in the PW-induced IRAK-M expression. These results provide better understanding of the Trp displaying nanodevices-induced IRAK-M expression and the subsequent effects on the TLR inhibition.

### The Therapeutic Efficacy of PW in LPS-Induced ALI Mouse Model

**Figure 7:**
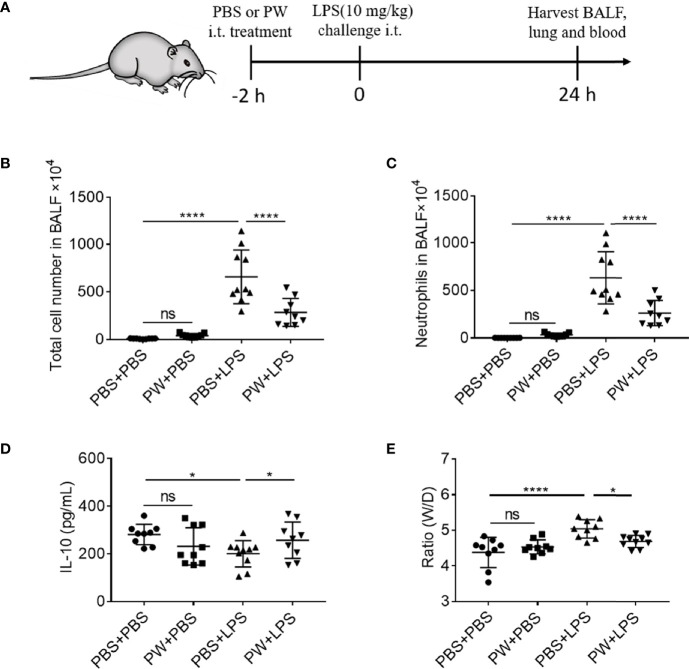
The inhibitory effect of PW on the lung inflammation in LPS-induced ALI mice. **(A)** The scheme of the LPS-induced ALI mouse model; PW (1.25 nmol/kg) was intratracheally administered 2 h before the LPS (10 mg/kg) challenge through the same route for 24 h; the BALF was collected for the analysis of the total number of cells **(B)** and the neutrophils **(C)** infiltrated in the lung. **(D)** The level of the anti-inflammatory cytokine IL-10 in the BALF. **(E)** The pulmonary edema assessed by the lung W/D ratio. N ≥ 9 per group; ns, not significant, *p < 0.05, ****p < 0.0001.

We have demonstrated that PW was able to inhibit TLR signaling pathways and exhibit anti-inflammatory activity *in vitro*. Next, we employed a mouse model of LPS-induced ALI to examine the therapeutic activities of PW *in vivo*. PW (1.25 nmol/kg) was administered by intratracheal injection 2 h before the LPS (10 mg/kg) challenge through the same route, and the bronchoalveolar lavage fluid (BALF) and lung tissues were collected for the analysis of lung inflammation and injury 24 h after LPS challenge (**Figure 7A**). We found that the PW treatment was able to reduce the LPS-induced lung inflammation by decreasing the total cell and neutrophil counts in the BALF (**Figures 7B, C**) and increasing the anti-inflammatory cytokine IL-10 in the BALF (**Figure 7D**), and reducing the ratio of wet-to-dry lung (W/D ratio) indicating the severity of pulmonary edema (**Figure 7E**). It is worth noting that PW treatment itself did not induce severe inflammatory responses *in vivo*. The cytospin images of the BALF cells revealed that PW was preferentially accumulated in the alveolar macrophages, indicating its targeting ability to macrophages (**Supplementary Figure S5**).

**Figure 8:**
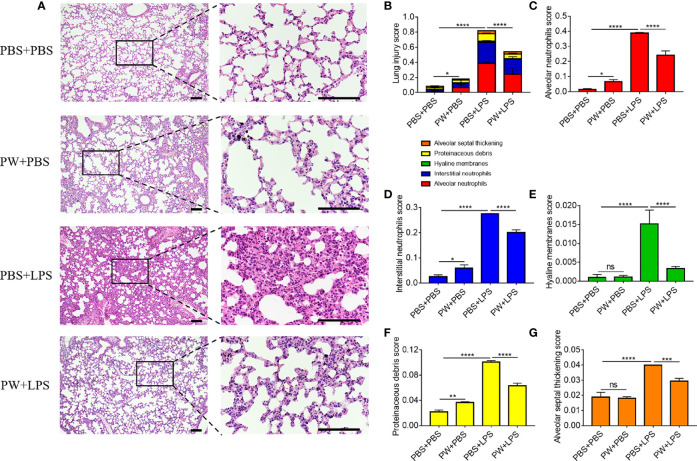
The protective effect of PW on the histopathological damages of the lung in LPS-induced ALI mice. **(A)** The images of lung sections stained with H&E; the scale bar = 100 µm. **(B)** The lung injury score based on the 5 pathophysiological characteristics: the alveolar neutrophils **(C)**, interstitial neutrophils **(D)**, hyaline membranes **(E)**, proteinaceous debris **(F)** and alveolar septal thickening **(G)**. N ≥ 9 per group; ns, not significant, *p < 0.05, **p < 0.01, ***p < 0.001, ****p < 0.0001.

The lung injury and inflammation were also evaluated by histopathological analyses of both peribronchiolar and perivascular inflammatory infiltration on H&E stained lung tissue sections (**Figure 8A**). PW treatment attenuated LPS-induced ALI as quantified by the injury scores of the 5 histological features (**Figure 8B**): the alveolar neutrophils (**Figure 8C**), interstitial neutrophils (**Figure 8D**), hyaline membranes (**Figure 8E**), proteinaceous debris (**Figure 8F**) and alveolar septal thickening (**Figure 8G**). Note that the PW treatment alone could slightly increase the injury score, especially the alveolar and interstitial neutrophils as well as the proteinaceous debris scores, but much less than the LPS group. Overall, PW was able to alleviate LPS-induced ALI in mice although the hybrid alone may prime some mild inflammatory responses in the lung.

In addition to LPS-induced ALI, we also established a mouse model of Poly I/C-induced acute lung inflammation to investigate the therapeutic effect of PW on excessive TLR3 activation. PW (1.25 nmol/kg) was given intratracheally 2 h before Poly I/C (2.5 mg/kg) challenge twice at 0 and 24 h through the same route; the BALF or lung tissue was collected 24 h after the second Poly I/C challenge (**Supplementary Figure S6A**). We found that the Poly I/C challenge caused moderate inflammatory infiltration (compared with the LPS challenge) to the lung, and the PW treatment was able to decrease the number of total cells and neutrophils in the BALF (**Supplementary Figures S6B, C**). Similarly, PW treatment could also reduce the lung injury score from the histopathological analyses (**Supplementary Figures S6D, E**). Specifically, the PW treatment reduced the number of alveolar and interstitial neutrophils, hyaline membrane, protein fragments and alveolar septum thickening (**Supplementary Figures S6F–J**). These results suggested that PW could alleviate TLR3 activation-mediated lung inflammation in mice, making PW a potent anti-inflammatory agent targeting multiple TLR signaling pathways for treating ALI.

## Discussion

Multiple lines of evidences have demonstrated that macrophages play a central role in the initiation, progression and resolution of inflammation in various disease conditions. Regulation of macrophage activation and its inflammatory responses would provide a promising therapeutic strategy to intervene such detrimental conditions. Nanodevices can naturally target macrophages through endocytosis and phagocytosis owing to their nanoscale property, making them a new class of versatile and effective agents to modulate the biological function of macrophages. In this study, we showed that a special class of Trp-containing hexapeptide-coated GNPs, PW, could trigger minute pro-inflammatory responses of macrophages; however, these nano-hybrids inhibited multiple TLR signaling pathways and exhibited potent anti-inflammatory activities *in vitro* and *in vivo* under the strike of strong inflammatory stimuli. This unique activity of PW could be attributed to the two arms of mechanistic actions: the blocking of endosomal acidification to inhibit TLR signaling, and the up-regulation of IRAK-M expression to dampen NF-κB activation in macrophages. The latter might contribute to the observed priming effect of PW for protection of macrophages from a subsequent insult by TLR stimulation in analogy to the known endotoxin tolerance effect. The Trp-containing peptide-GNP hybrids by design represented a novel immunomodulatory nanodevice that could be applied to manage the dysregulated innate immune responses for treating inflammatory conditions as in ALI and its severe form of acute respiratory distress syndrome (ARDS).

### Nanodevice-Based Immune Modulation of Macrophages

TLRs are one major class of pathogen recognition receptors (PRRs) responsible for mounting innate immune responses in the host defense against infections or non-infectious insults (25). Excessive activation of TLR is associated with many inflammatory disorders, and hence regulating TLR signaling has become an attractive intervention strategy. Herein, we discovered that a peptide-decorated nanodevice (PW) that displays tryptophan residues on the surface could inhibit multiple TLR pathways including TLR4, TLR3 and TLR7/8 (**Figures 2** and **3**), and the production of pro-inflammatory cytokines (IL-6, IL-12/IL-23p40, TNF-α and MCP-1). Interestingly, PW did not affect TLR2 signaling, suggesting that PW preferentially acted on endosomal-related TLR signaling. The ability of inhibiting multiple TLR pathways makes PW a potent nano-inhibitor to regulate multifactorial, overwhelming inflammatory reactions in complex diseases like ALI/ARDS.

It was surprising to us that PW alone had mild pro-inflammatory activity on NF-κB activation (**Figures 2**, **4**). This unique property of PW appeared to rely on the tryptophan residues displaying on the nanodevice. When replacing WW residues in the peptide coating on PW to either other hydrophobic amino acids (FF, LL, II, AA) or hydrophilic ones (SS, TT), these nano-hybrids did not activate NF-κB (**Figure 6H**). Moreover, neither the peptides alone nor the bare GNPs induced the activation of NF-κB (**Supplementary Figure S1**). Thus, the specific presence of the membrane anchoring amino acid tryptophan on the GNP surface imparts the nano-hybrid novel activity to mildly activate macrophages. Such an action on the contrary primed macrophages to lower the response for the subsequent inflammatory stimulation (see below IRAK-M).

On the other hand, the uptake of PW in macrophages could significantly modulate the endosomal pH, which in turn inhibited the TLR signaling (**Figure 5**). It has been found that the acidification process of endosomes/lysosomes can regulate many signaling events (26). For TLR4 signaling, the TRIF-dependent signal transduction requires the trafficking of TLR4 from the cell surface to the endosomes/lysosomes (27). During the trafficking process from early endosomes to late endosomes or lysosomes, the microenvironment changes accordingly including acidification in order to convey the signals to trigger corresponding cellular responses. Blockade of the endosomal/lysosomal acidification process is thus expected to affect the endosomal TLR signaling. PW would presumably behave like our previously developed anti-inflammatory nanoparticle P12, which can act like a proton sponge to sequester protons due to the negative charge of the aspartate (with side chain pKa of ~3.9) on the nanoparticles, consequently blocking the normal acidification process in the endosomes/lysosomes and inhibiting the endosomal TLR signaling.

Different from small molecule inhibitors, PW-based nanodevices have many advantages on TLR signaling modulation for basic and translation research. First, these nanodevices have targeting capability to phagocytic immune cells. Second, they can be easily traced based on the characteristics of the GNPs. Third, they can have preferred biodistribution and pharmacokinetic profiles by design. More importantly, the priming effect of PW and its TLR inhibitory activity resemble many phenomena reported in trained immunity, where exogenous or endogenous stimuli can prime the immune system to lunch a proper response (stronger or weaker) to the second attack (28). Therefore, these nanodevice-based TLR modulators could provide novel ideas to train our immune system to manage the detrimental inflammatory responses in many diseases.

### The Negative Regulator IRAK-M in TLR Signaling and the Potential Mechanism(s) of PW-Mediated Priming Effects on TLR Inhibition

IRAK-M, a member of the IRAK family lacking kinase activity, is one of the important negative regulators of TLR signaling (20). It is mainly expressed in myeloid cells and regulates the immune homeostasis and tolerance (29, 30). IRAK-M negatively regulates NF-κB activation by competitively binding to IRAK1/4 to block the kinase activity, and hence consequently inhibits the down-stream signaling cascades (31). Therefore, the induction of IRAK-M expression could limit the pathological damages caused by overactivation of the inflammatory signaling. In our studies, we found that PW could particularly elevate IRAK-M expression in macrophages (**Figure 6**), which was governed by the tryptophan (W) residues on the nanodevices as replacing the two tryptophan (WW) residues in PW with other amino acids (FF, LL, II, AA, SS and TT) abolished such an effect (**Figures 6E–G**). The up-regulation of IRAK-M by PW may explain the observed priming effect of PW on the TLR inhibition. In fact, mice with IRAK-M deficiency exhibited enhanced inflammatory responses to infection (32). Furthermore, compared with wild-type mice, the IRAK-M knockout mice had more inflammatory cell infiltration and higher pro-inflammatory cytokine production in the lung in response to OVA challenge (33). These evidences suggest that IRAK-M is essential in maintaining the immune homeostasis during inflammatory responses.

The expression of IRAK-M in macrophages can be induced by various endogenous and exogenous soluble factors, as well as inter- or intracellular signaling molecules. These molecules include molecular patterns of pathogen products such as LPS, flagellin, peptidoglycan (PGN) and CpG (34). Actually, IRAK-M induction is a very common phenomenon in endotoxin tolerance, a protective mechanism in which cells or organisms enter into a transient unresponsive state upon exposure to low dose of endotoxin, so they are unable to respond to a second challenge of endotoxin (17). Although many other factors, such as prostaglandin E2 (PGE2), GM-CSF, IL-13, pulmonary surfactant protein A and surfactant lipids as well as glucocorticoids, can up-regulate the expression of IRAK-M in macrophages (35, 36), currently to our knowledge there is no report on nanoparticle-induced IRAK-M expression except PW in our study (**Figure 6**).

Although the entry of PW into macrophages was primarily through micropinocytosis (**Figure 5B**), we found that the mannose receptor (MR) was involved in PW-induced IRAK-M expression. It has been found that a protein toxin released from the gram-positive bacteria of *Streptococcus pneumoniae*, pneumolysin (PLY), interacts with the mannose receptor depending on its tryptophan motif (23, 24). Binding of PLY to the mannose receptor C type 1 (MRC-1/CD206) on the mouse alveolar macrophages can reduce TLR signaling and pro-inflammatory cytokine production as well as infiltration of neutrophiles to the lung (24). Other studies also reported that the MR agonists, mannose-capped lipoarabinomannans (Man-LAMs), could inhibit LPS-induced IL-12 production through IRAK-M induction in mouse macrophages (37, 38). Herein, we showed that PW-mediated IRAK-M expression in macrophages was dependent on the tryptophan residues, and the MR was involved in the phenomenon (**Figures 6E–L**). More experiments with genetic tools are required in the future to confirm the specific role of IRAK-M induction in the observed TLR inhibition and anti-inflammatory activities of PW.

### Trp-Displaying Nanodevices as a New Type of Immunomodulatory Nanotherapeutics for Treating ALI/ARDS

ALI/ARDS is a life-threatening condition with respiratory failure characterized by uncontrolled, rapid, widespread inflammation in the lungs (39). There are currently no effective pharmacological treatments for ALI/ARDS. Studies have shown that in the early stage of ALI/ARDS, alveolar macrophages (AM) and pattern recognition receptors (PRRs) on the AM surface, especially TLRs, contribute to the initiation of inflammatory responses (40, 41). Therefore, effective regulation of TLR signaling of lung macrophages may provide a promising strategy to treat ALI/ARDS. In this study, the developed PW could specifically target lung macrophages to attenuate TLR signaling and decrease pro-inflammatory cytokine production in two ALI mouse models (LPS and Poly I/C challenge) (**Figures 7**, **8**, and **Figure S6**). The potent inhibitory activity of PW on multiple TLR pathways, together with its macrophage targeting ability and tryptophan-specific regulatory function makes PW a promising therapeutic agent to treat ALI/ARDS. Nevertheless, PW is not biodegradable, and the development of new forms of PW is required for future clinical translation.

## Conclusions

In conclusion, we developed a Trp-displaying nanodevice, PW, with unique immunomodulatory activities. PW was made by modifying GNPs with a peptide containing two Trp residues in the sequence. PW itself induced mild pro-inflammatory responses but exhibited potent anti-inflammatory activities with the presence of inflammatory stimuli through inhibiting multiple TLR (3, 4 and 7/8) signaling cascades in macrophages. This inhibitory activity was primarily attributed to the modulation of the endosomal pH and hence preferentially affecting the endosomal TLR signaling. Very interestingly, the PW alone could induce the expression of the negative regulator IRAK-M in the TLR signaling, which depended on the presence of Trp residues. The up-regulation of IRAK-M may contribute to the priming effect of PW on the inhibition of subsequent stimulation by LPS. The therapeutic effects of PW were assessed on two mouse models of LPS- and Poly I/C-induced ALI. It was found that PW pre-treatment was able to reduce the inflammatory cells infiltration, particularly neutrophils, and increase the anti-inflammatory cytokine IL-10 level in the BALF as well as decrease the lung injury and edema. This study defined a new design principle of using the membrane anchoring amino acid Trp to enable nanodevice-based TLR inhibitors with novel immunomodulatory capability, which served as a new class of anti-inflammatory therapeutics for treating inflammatory diseases such as ALI/ARDS.

## Data Availability Statement

The original contributions presented in the study are publicly available. This data can be found here: https://www.ncbi.nlm.nih.gov/geo/query/acc.cgi?acc=GSE181851.

## Ethics Statement

The animal study was reviewed and approved by The Animal Care and Use Committee at Tianjin Medical University.

## Author Contributions

HY and SYF conceived the study and designed the research. HY, SYF, and LS wrote the manuscript. LS synthesized and purified the nanoparticles, and assessed their anti-inflammatory effects *in vitro* and *in vivo*. RW performed RNA-seq experiment and analysis. CW assisted cell culture and the animal experiments. JG conducted confocal microscopy experiments. HM performed TEM imaging. LS, RW, and JG analyzed the results and generated the figures. All authors contributed to the article and approved the submitted version.

## Funding

This work was supported by the National Natural Science Foundation of China (No. 81770070 for HY and No. 81971549 for SYF), the Natural Science Foundation of Tianjin Municipal Science and Technology Commission (20JCYBJC00040 for HY) and the starting fund from Tianjin Medical University.

## Conflict of Interest

The authors declare that the research was conducted in the absence of any commercial or financial relationships that could be construed as a potential conflict of interest.

## Publisher’s Note

All claims expressed in this article are solely those of the authors and do not necessarily represent those of their affiliated organizations, or those of the publisher, the editors and the reviewers. Any product that may be evaluated in this article, or claim that may be made by its manufacturer, is not guaranteed or endorsed by the publisher.
