# Supplementary material for: The Modulatory Activity of Tryptophan Displaying Nanodevices on Macrophage Activation for Preventing Acute Lung Injury
Source: Front Immunol. 2021 Sep 30;12:750128. doi: 10.3389/fimmu.2021.750128 (PMC8516359; doi:10.3389/fimmu.2021.750128)
Supplement: Supplementary file 1 [file DataSheet_1.pdf]

# Supplementary Material

## 1 Supplementary Methods

### 1.1 Reporter cells assay for the analysis of NF-κB/AP-1 activation of macrophages

THP-1-XBlue reporter cells were seeded into a 96-well flat plate and differentiated into macrophages. Cells were treated with CLPWWD at various concentrations (150, 100 and 50 μM) or bare 13-nm GNPs (GNP13, 100 nM) in the presence and absence of LPS (10 ng/mL) for 24 h. For the JNK inhibitor SP600125 study, cells were treated with PW (100 nM) in the presence and absence of SP600125 (10 μM) for 24 h. Culture media were collected for the analysis of NF-κB/AP-1 activation by QUANTI-Blue assay. The color change was quantified by the absorption measurement at 655 nm on a microplate reader (Spark, TECAN, Mannedorf, Zurich, Switzerland).

### 1.2 Immunoblotting analysis

THP-1 monocytes ($5\times10^5$ cells/well) were seeded into a 24-well plate and differentiated into macrophages. Cells were treated with mannan at various concentrations (50, 10 and 5 μg/mL) or PW (100 nM) for 24 h, and then were lysed in ice-cold RIPA lysis buffer. The immunoblotting analysis of the lysed samples were performed following the same procedure described in the Methods section of the main text.

### 1.3 Acute lung injury murine model stimulated by Poly I/C

C57BL/6 wild-type male mice (6-8 weeks from SPF Biotechnology Co., Ltd, Beijing, China) were used to generate the ALI mouse model by intratracheal administration of Poly I/C (HMW). After intraperitoneal injection of 1% sodium pentobarbital anesthesia (45 mg/kg), PW (1.25 nmol/kg) was intratracheally administered, and Poly I/C (2.5 mg/kg) was given through the same route 2 h later. The mice were challenged with Poly I/C (2.5 mg/kg) for the second time after the first challenge for 24 h, and the mice were sacrificed for lung inflammation and injury analysis at 50 h post PW treatment.

## 2 Supplementary Figures

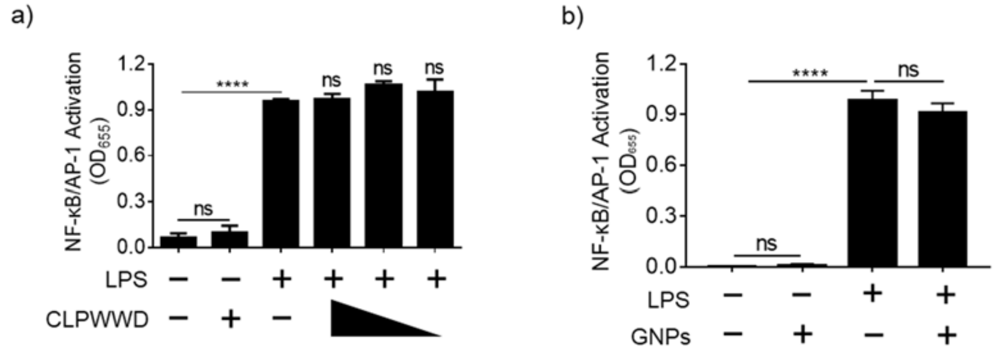


**Supplementary Figure 1.** The effect of the peptide alone (a) and the bare GNP (b) on the activation of NF-κB/AP-1 in the THP-1 reporter cell-derived macrophages in the absence or presence of LPS (10 ng/mL). The peptide concentrations: 50, 100 and 150 μM (with LPS), and 150 μM in the absence of LPS; the bare GNP: 100 nM. ns: not significant vs. LPS, ****p < 0.0001.

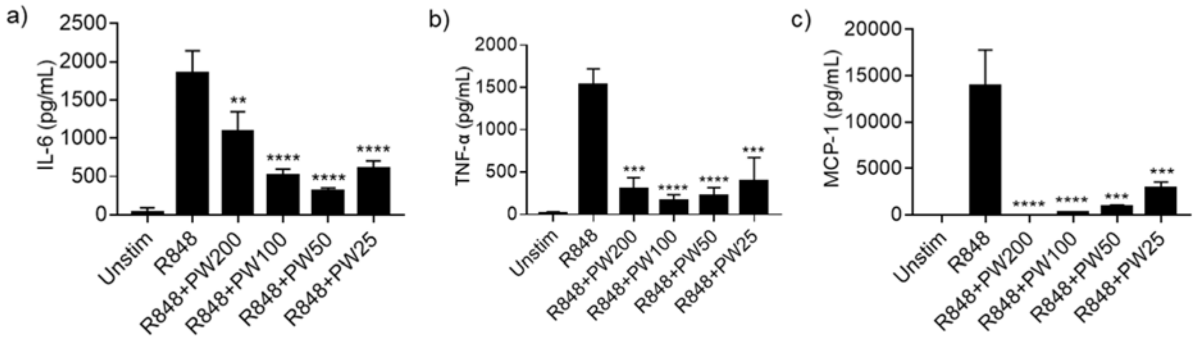


**Supplementary Figure 2.** Inhibition of R848-induced IL-6 (a), TNF-α (b) and MCP-1 (c) production by PW treatment in THP-1 monocytes. N ≥ 3; R848: 10 μg/mL; PW: 200, 100 and 50 nM. **p < 0.01, ***p < 0.001, ****p < 0.0001.

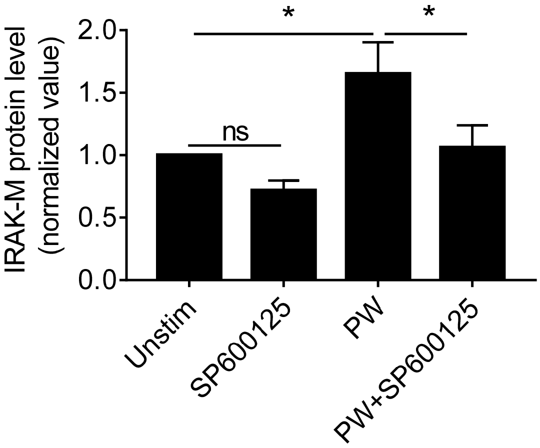


**Supplementary Figure 3.** Quantitative analysis of the inhibition of PW-induced IRAK-M expression by SP600125 in macrophages from the immunoblots in Figure 6j. SP600125 = 10 μM, *$p < 0.05$, ns: not significant.

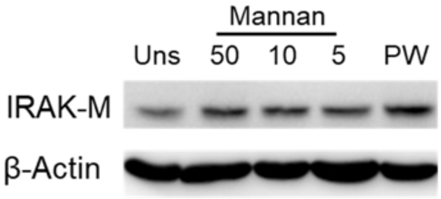


**Supplementary Figure 4.** The concentration effect of mannan on IRAK-M expression. Cells were treated with mannan or PW for 24 h. The concentration of mannan = 50, 10 or 5 μg/mL; PW = 100 nM. β-actin as the internal control.

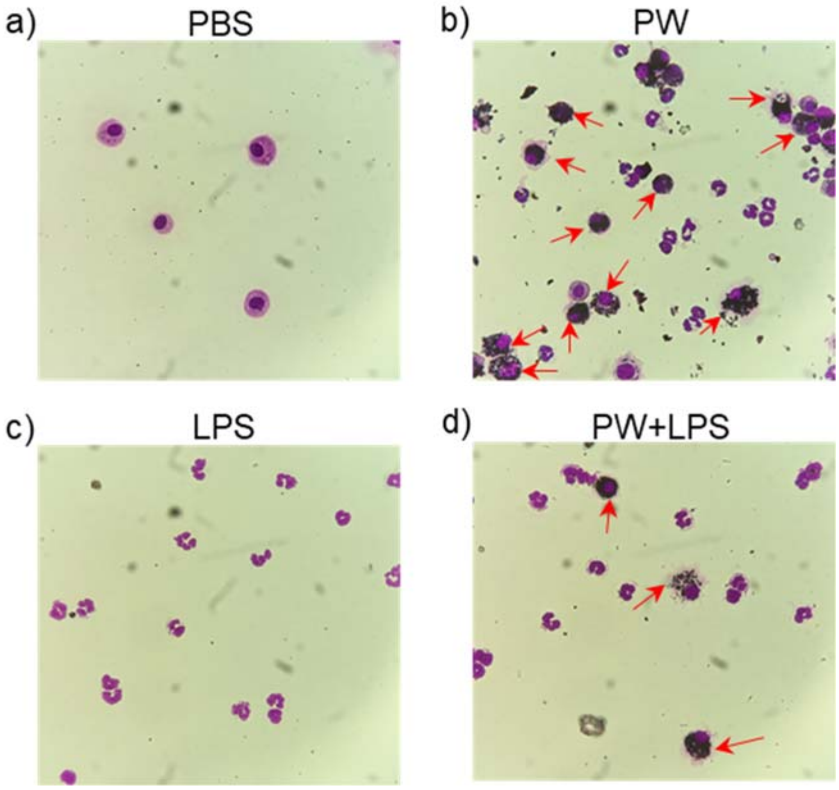


**Supplementary Figure 5.** Liu staining of infiltrated cells in the BALF collected from the four experimental groups: (a) PBS+PBS, (b) PW only, (c) PBS+LPS, and (d) PW+LPS. The red arrows indicate alveolar macrophages with large amount of PW uptake (dark blue or black dots in macrophages). These photographs show that PW passively targets the alveolar macrophages.

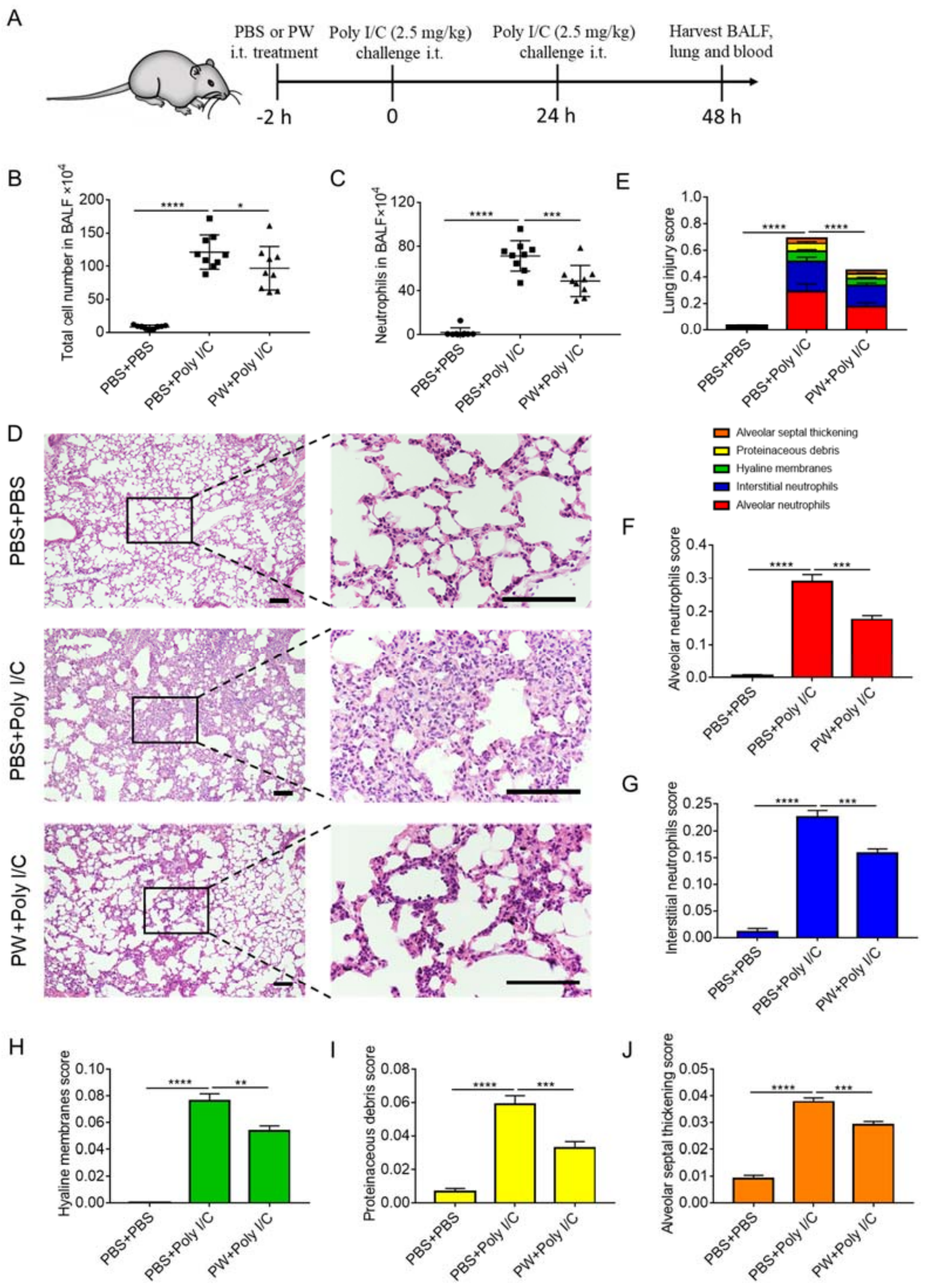


**Supplementary Figure 6.** The protective effect of PW on the lung damages in a Poly I/C-induced ALI mouse model. (a) The scheme showing the PW treatment in the Poly I/C-induced ALI model. (b) The total number of infiltrated cells in the BALF. (c) The neutrophil counts in the BALF. (d) The images of H&E stained lung sections of the three experimental groups (PBS+PBS, PBS+Poly I/C and PW+Poly I/C) 24 h after Poly I/C stimulation. The scale bar = 100 μm. (e) The total lung injury score obtained from the 5 pathophysiological characteristics: (f) alveolar neutrophil, (g) interstitial neutrophil, (h) hyaline membrane, (i) protein debris score, and (j) alveolar septal thickness. PW: 1.25 nmol/kg; N ≥ 6 per group; $*p < 0.05$, $**p < 0.01$, $***p < 0.001$, $****\ p < 0.0001$.
